# Comparison of Gd-EOB-DTPA-enhanced MRI versus multiphasic enhanced CT for detecting occult recurrence after curative ablation of hepatocellular carcinoma

**DOI:** 10.3389/fonc.2026.1767884

**Published:** 2026-03-12

**Authors:** Zhi Zhu, Mingyu Zhang, Fengcan Cai, Yuanyong Zhou, Qingde Wu

**Affiliations:** 1 Shunde Hospital Guangzhou University of Traditional Chinese Medicine, Foshan, China; 2 Guangzhou University of Traditional Chinese Medicine, Guangzhou, China

**Keywords:** computed tomography, gadoxetate disodium, hepatocellular carcinoma, magnetic resonance imaging, microwave ablation, neoplasm recurrence

## Abstract

**Objective:**

To evaluate the value of Gd-EOB-DTPA-enhanced MRI in the early diagnosis of occult intrahepatic recurrence after curative ablation for hepatocellular carcinoma (HCC).

**Materials and methods:**

This retrospective study included 74 HCC patients who underwent curative microwave ablation, divided into a CT group and a CT+MRI group. An expert committee determined recurrence via consensus readout. Diagnostic accuracy, sensitivity, specificity, positive predictive value (PPV), negative predictive value (NPV), and the area under the receiver operating characteristic curve (AUROC) of the two imaging methods were compared through blinded image review.

**Results:**

With a median follow-up of 23.5 months, 38 patients (51.4%) were diagnosed with recurrence. Within the CT+MRI group, the sensitivity of Gd-EOB-DTPA-enhanced MRI for diagnosing recurrent patients was significantly higher than that of enhanced CT (P < 0.05), leading to altered BCLC stages in 2 patients. Low signal intensity on the hepatobiliary phase was the most sensitive finding. Diagnostic performance analysis confirmed that the AUROC and sensitivity of Gd-EOB-DTPA-enhanced MRI were significantly superior to those of enhanced CT (all P < 0.05).

**Conclusion:**

Gd-EOB-DTPA–enhanced MRI demonstrated superior diagnostic performance compared with multiphasic contrast-enhanced CT for detecting CT-occult intrahepatic recurrence after curative MWA. However, because MRI was selectively performed in patients with high-risk features, elevated AFP, or equivocal CT findings, its benefit is best interpreted as an adjunctive tool in this targeted population rather than a routine replacement for CT surveillance. A risk-stratified follow-up strategy may optimize clinical benefit and cost-effectiveness.

## Introduction

Microwave ablation (MWA), a minimally invasive and repeatable modality, is widely employed as a local curative treatment for unresectable hepatocellular carcinoma (HCC). Nonetheless, intrahepatic recurrence occurs in over 60.0% of patients within five years after curative ablation (1, 2). Furthermore, there is a current lack of adjuvant targeted-immunotherapy regimens demonstrating definitive long-term survival benefits (3, 4). Consequently, post-procedural imaging surveillance is critical for the timely and accurate diagnosis of recurrence. This assessment directly determines whether a patient is eligible for repeated curative therapy (e.g., repeat ablation or resection) or requires a transition to palliative options (e.g., TACE or systemic therapy), thereby significantly impacting long-term survival and quality of life (5, 6). Although contrast-enhanced computed tomography (CECT) is the standard modality for post-treatment surveillance, recurrent HCC often exhibits atypical enhancement patterns compared to primary tumors. This is frequently due to smaller lesion size (≤2 cm) and immature tumor vasculature. This makes recurrence inconspicuous against a cirrhotic background and limits the sensitivity of CECT, leading to missed diagnoses (7).

In contrast, liver MRI utilizes the benefits of multiparametric imaging to not only clearly display vascular structures but also assess tissue cellularity through diffusion-weighted imaging (DWI) without reliance on lesion blood supply, thereby demonstrating superior clinical value in evaluating atypically enhancing lesions. However, the diagnostic accuracy of conventional MRI for lesions ≤2.0 cm remains suboptimal (8, 9). Gadolinium-ethoxybenzyl-diethylenetriamine pentaacetic acid (Gd-EOB-DTPA) is a hepatocyte-specific MRI contrast agent with dual properties for both hemodynamic and hepatobiliary phase imaging. During the hepatobiliary phase (HBP), its high uptake by functioning hepatocytes causes marked hyperintensity of the background liver parenchyma, creating a stark contrast with lesions that lack uptake (10, 11). This property gives gadoxetate-enhanced MRI superior diagnostic performance in detecting small (≤2 cm) lesions (12, 13). Studies have shown that in patients with Barcelona Clinic Liver Cancer (BCLC) stage 0 or A HCC, utilizing gadoxetate-enhanced MRI as an adjunctive imaging tool can identify additional HCC foci, leading to modifications in treatment strategy and subsequently improving recurrence-free and overall survival rates (14).

Nevertheless, compared to the standard follow-up with contrast-enhanced CT, the value of gadoxetate-enhanced MRI for detecting occult intrahepatic recurrence after HCC treatment remains uncertain. Hence, this retrospective study was designed to compare the diagnostic performance of Gd-EOB-DTPA-enhanced MRI and multiphase contrast-enhanced CT in identifying occult intrahepatic recurrence following curative MWA for HCC.

## Methods

This single-center, retrospective study was performed in compliance with the Declaration of Helsinki. The institutional ethics review committee approved the protocol and granted a waiver of informed consent based on the retrospective design. Given the non-randomized allocation of groups, a dual analytical strategy was adopted for a comprehensive assessment of MRI’s value. This consisted of, first, an intra-group paired analysis to directly compare CT versus MRI diagnostic performance, followed by an inter-group comparison of the diagnostic performance differences between the two modalities.

A retrospective analysis was conducted on HCC patients who underwent MWA at our institution between January 2018 and January 2024. Inclusion criteria for the study were (1): Diagnosis of HCC confirmed according to EASL guidelines, either pathologically and/or by non-invasive criteria (i.e., typical imaging features of HCC on 4-phase contrast-enhanced CT, dynamic contrast-enhanced MRI, or CEUS) (3); (2) All tumor nodules identified on pre-ablation imaging were completely ablated; (3) Availability of complete pre-procedural imaging and regular follow-up images on at least three occasions post-procedure. Exclusion criteria were (1): History of other concurrent malignancies; (2) Inadequate follow-up time (less than 6 months); (3) Poor image quality insufficient for diagnosis or missing crucial image data.

All patients at our center closely adhered to guideline-recommended follow-up protocols. Surveillance included serum alpha-fetoprotein (AFP) testing and regular imaging studies—every 3 months for the first 2 years after treatment, and every 3–6 months thereafter—with follow-up continued until January 2025.

Patients were assigned to the CT-only group when they underwent routine surveillance exclusively with multiphasic contrast-enhanced CT and did not meet the criteria for supplemental MRI evaluation throughout the follow-up period. Patients were assigned to the CT+MRI group when, during follow-up, they underwent a contemporaneous Gd-EOB-DTPA-enhanced MRI examination in addition to CT, performed within one week of a surveillance CT. The clinical indications for performing supplemental MRI included (1): presence of high-risk features for recurrence (multiple initial tumors, maximum tumor diameter ≥ 5 cm, or poor tumor differentiation on initial pathology) (2); inconclusive or equivocal findings on surveillance CT that could not definitively exclude recurrence (3); elevated or rising serum AFP levels despite negative or inconclusive CT findings; or (4) high clinical suspicion of recurrence based on overall clinical assessment. Importantly, no patient in our cohort underwent Gd-EOB-DTPA-enhanced MRI for routine surveillance in the absence of these indications.

### Diagnosis of postoperative recurrence

The diagnosis of HCC recurrence was adjudicated by a committee consisting of three senior hepatobiliary radiologists (all holding the position of chief physician with 15–20 years of experience). Through a consensus review process, the committee determined the intrahepatic recurrence status for each patient. All recurrence diagnoses were required to be confirmed either by subsequent follow-up imaging demonstrating definite lesion growth or by post-treatment verification of a viable tumor.

Occult recurrence was defined as an intrahepatic lesion occurring after curative ablation that was either missed or could not be confidently characterized as recurrence on multiphasic contrast-enhanced CT during follow-up, but was detectable on contemporaneous Gd-EOB-DTPA–enhanced MRI. These lesions were subsequently confirmed as true recurrence based on follow-up imaging evolution (interval growth and/or progression toward a typical malignant enhancement pattern) and/or imaging response after salvage therapy. Notably, the term “occult” in this study refers to CT-occult but MRI-detectable recurrence, rather than lesions invisible on all imaging modalities.

### Blinded evaluation of diagnostic performance

To further assess diagnostic performance, a blinded image review was conducted. Based on the on-site consensus reading results, the diagnostic accuracy, sensitivity, specificity, positive predictive value (PPV), and negative predictive value (NPV) of two imaging modalities (Gd-EOB-DTPA-enhanced MRI versus multiphase enhanced CT) were compared at both the patient-level and lesion-level. This process involved two observers (Observer 1 and Observer 2 with 5 and 10 years of hepatobiliary imaging experience, respectively), who were independent of the adjudication committee. For the blinded reader study, Observer 1 and Observer 2 independently reviewed CECT and gadoxetate-enhanced MRI examinations. CT and MRI were interpreted separately rather than side-by-side to avoid cross-modality influence. Disagreements between the two observers were not resolved by consensus for the purpose of diagnostic performance calculation; instead, interobserver agreement was quantified using Cohen’s kappa. The reference standard for recurrence was the independent adjudication committee consensus with follow-up confirmation.

To ensure consistency and accuracy in the evaluation, the two observers were provided with the following standardized criteria prior to the formal assessment (15–17): A nodule exhibiting the typical “wash-in and wash-out” enhancement pattern (i.e., arterial phase hyperenhancement with subsequent washout in the portal venous or delayed phase) on CT or MRI could be directly diagnosed as HCC recurrence;For nodules with atypical enhancement features,CT diagnosis required meeting both of the following criteria, hypodensity in the portal venous or delayed phase, and features suggestive of malignant infiltration (e.g., ill-defined borders, irregular shape),MRI diagnosis required meeting at least three of the following four features: hypointensity in the portal venous or delayed phase, mild hyperintensity on T2-weighted imaging, hyperintensity on high b-value (1000 s/mm²) DWI, and hypointensity in the hepatobiliary phase.

### Ablation procedure planning

The overall ablation strategy was determined through pre-procedural multidisciplinary team (MDT) assessment of the tumor, while also considering patient preference and financial capacity. To achieve complete tumor ablation, the decision to perform pre-ablation transarterial chemoembolization (TACE) was based on liver function, tumor size (>5 cm), and location. All percutaneous MWA procedures were performed under local anesthesia with CT guidance by two interventional radiologists, each possessing over ten years of experience in HCC ablation. The ablation approach, power (typically 50-65W), and duration (typically 5–10 minutes) were tailored according to the size of each lesion. Staged ablation was performed in cases of multiple or geographically separated lesions, or when the patient’s physical condition precluded single-session ablation of all targets. Priority was given to ablating larger, hypervascular, and more threatening lesions first. An interval of two weeks was maintained between ablation sessions to achieve complete tumor destruction.

### Imaging methods

All patients underwent multiphase contrast-enhanced abdominal CT scans using a third-generation dual-source CT scanner (SOMATOM Force, Siemens Healthineers, Germany). Images were acquired with the following parameters: tube voltage 120 kV; tube current 280 mA; pitch 0.9-1; rotation time 0.40-0.80 s; field of view (FOV) 300–350 mm; matrix 512×512; slice thickness 5 mm. Iodinated contrast agent (1.2-1.5 ml/kg) was administered intravenously via an antecubital vein at a flow rate of 3–4 ml/s. Non-enhanced, arterial, portal venous, and delayed phase images were obtained before the injection and at 30–40 s, 55–65 s, and 155–165 s after the initiation of contrast injection, respectively. No changes in CT technology or instrumentation have occurred since January 2018.

MRI examinations for all patients were performed using a Siemens VIDA 3.0T MRI system (Germany) equipped with a 16-channel phased-array coil (T1vibe dicon sequence). The FOV was 350mm×350mm, matrix size 224×320, slice thickness 5.0 mm, and slice gap 1.0 mm. Gadoxetate disodium (Gd-EOB-DTPA) was injected at a dose of 0.025 mmol/kg via an antecubital vein at a flow rate of 2 mL/s, followed by a 20 mL saline flush at the same rate. Six dynamic phases were acquired at approximately 15, 30, 40, 50, 80, and 90 seconds post-injection, with a delayed phase obtained after 180 s. Diffusion-weighted imaging (DWI) was performed with b-values of 0, 50, and 1000 s/mm², using an FOV of 420 mm×420 mm, matrix size 118×148, and slice thickness 5.0 mm. The timing for the hepatobiliary phase scan was adjusted based on the patient’s liver function: it was initiated at 10 minutes post-injection for patients with normal liver function, at 15 minutes for those with mild hepatic impairment, and delayed up to 40 minutes (but not exceeding 60 minutes) for patients with chronic liver disease or cirrhosis.

### Statistical analysis

Statistical analyses were performed using SPSS software (version 25.0; IBM Corp.). The normality of continuous variables was assessed using the Shapiro-Wilk test. Normally distributed quantitative data are presented as mean ± standard deviation ( $\bar{\text{x}}$ ± s), non-normally distributed data as median (Q1, Q3), and categorical data as numbers and percentages. For comparing baseline characteristics between groups, independent samples t-tests or Mann-Whitney U tests were used for continuous variables based on their distribution, while chi-square or Fisher’s exact tests were applied for categorical variables. Diagnostic performance was evaluated by calculating the area under the receiver operating characteristic curve (AUROC) for paired comparisons, with differences between AUROCs assessed using DeLong’s test. Sensitivity, specificity, positive and negative predictive values, and diagnostic accuracy are reported with 95% confidence intervals (CI). Differences in these metrics were compared using McNemar’s test or Fisher’s exact test, as appropriate. Inter-group comparisons for categorical data were performed using chi-square or Fisher’s exact tests. All statistical tests were two-sided, and a P-value of less than 0.05 (P <.05) was considered statistically significant.

## Result

### Patient baseline characteristics

A total of 74 HCC patients who underwent MWA were included in this study. Based on the follow-up strategy, patients were divided into two groups: 37 patients received only multiphase enhanced CT during follow-up (CT group), while the other 37 patients underwent both multiphase enhanced CT and Gd-EOB-DTPA-enhanced MRI within a one-week interval (CT+MRI group). There were no statistically significant differences (P >.05) in key baseline characteristics between the two groups, including age, gender, initial tumor number, maximum tumor diameter, Child-Pugh grade, and AFP levels, indicating comparability (**Table 1**). The median follow-up time was 23.5 months (IQR 15.75, 37.25), with no significant difference between the groups (P = .922).

**Table 1:** Baseline and cohort characteristics of the 74 patients.

Characteristic	Total	CT	CT+MRI	Z	P
Mean age(IQR),y	62(57.8,67.0)	65(59.0, 69.5)	60(57.0,60.0)	1.597	0.110
Sex				0.140	0.708
Male	66(89.2)	32(88.4)	34(91.9)		
Female	8(10.8)	5(13.5)	3(8.1)		
Etiology				2.867	0.239
HBV	59(79.7)	29(78.4)	30(81.1)		
HCV	2(2.7)	2(5.4)	0 (0)		
Others	13(17.6)	6(16.2)	7(18.9)		
Cirrhosis	68(91.9)	35(94.6)	33(89.2)	0.181	0.670
Child-pugh				2.525	0.112
A	67(90.5)	31(83.8)	36(97.3)		
B	7(9.5)	6(16.2)	1(2.7)		
BCLC				0.000	1.000
0	25(33.8)	13(35.1)	12(32.4)		
A	49(66.2)	24(64.9)	25(67.6)		
Median α-fetoprotein level(IQR), ng/Ml	13.5(5.0,130.8)	13(4.5,101.4)	14(5.5,329.0)	0.850	0.395
Median albumin level(IQR), g/dL	41(36.4,44.6)	41(36.0,43.0)	41(37.3,46.0)	0.975	0.330
Median total bilirubin level(IQR), mg/dL	12.3(8.7,16.0)	13(8.5,16.0)	11(8.5,14.5)	0.964	0.335
Median platelet count(IQR), ×1000/mm^3^	139(98.0,184.8)	134(93.5,176.5)	141(119.0,188.5)	1.000	0.317
Median AST level(IQR), IU/mL	34.3(27.0,47.9)	36(28.5,50.0)	34(26.5,45.2)	0.735	0.462
Median ALT level(IQR), IU/mL	31.8(17.8,39.6)	34(19.0,43.8)	29(17.2,37.7)	0.827	0.408
Median size of primary HCC(IQR), cm	2.6(1.6,6.0)	2.3(1.8,4.9)	2.8(1.5,7.5)	0.590	0.555
Number of Recurrences	38(51.4)	18(48.6)	20(54.1)	0.216	0.642

AST, aspartate aminotransferase; ALT, alanine aminotransferase; HBV, hepatitis B virus; HCV, hepatitis C virus; IQR, interquartile range.

### Diagnosis of HCC recurrence

Among the 74 patients, 38 (51.4%) were diagnosed with intrahepatic HCC recurrence via on-site consensus reading. This included 18 patients in the CT-alone group and 20 patients in the CT+MRI group. Of the 38 patients with recurrence, 2 exhibited diffuse intrahepatic recurrence. The remaining 36 patients had a total of 41 recurrent HCC lesions identified, with a median diameter of 1.8 cm (IQR 1.4, 2.8).

### Impact of Gd-EOB-DTPA-enhanced MRI on the detection of recurrent HCC and BCLC staging

**Figure 1:**
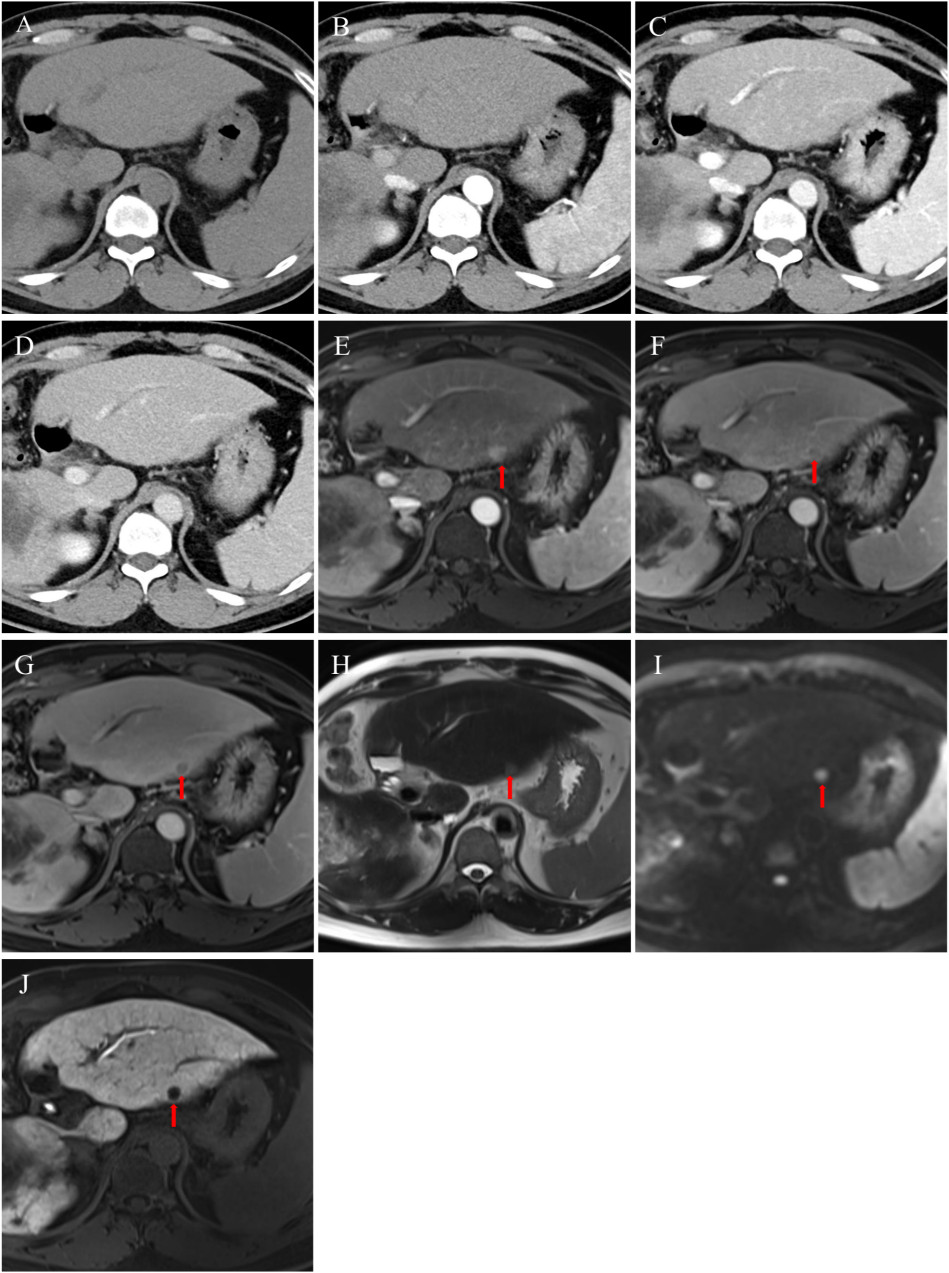
A case of a 50-year-old male patient with a history of hepatitis B-related cirrhosis, presenting with 11-month follow-up imaging after curative microwave ablation (MWA) for hepatocellular carcinoma (HCC). Initial multiphase contrast-enhanced CT **(A–D)** showed no definite signs of intrahepatic recurrence. However, comparative analysis of the same level using an additionally performed Gd-EOB-DTPA-enhanced MRI **(E–J)** revealed a approximately 1.3 cm lesion in segment III of the left hepatic lobe. The imaging characteristics of this lesion were consistent with HCC: arterial phase hyperenhancement **(E)**, "washout" appearance (hypointensity) in the portal venous **(F)** and delayed phases **(G)**, mild hyperintensity on T2-weighted imaging **(H)**, restricted diffusion on diffusion-weighted imaging **(I)**, and hypointensity on the hepatobiliary phase **(J)**. The lesion was ultimately confirmed as an intrahepatic HCC recurrence.

In the patient-based analysis for the CT+MRI group, Gd-EOB-DTPA-enhanced MRI correctly diagnosed all 20 (100%) patients with recurrence. In contrast, the contemporaneous multiphase enhanced CT correctly identified only 14 (70%) of these patients. Consequently, Gd-EOB-DTPA-enhanced MRI detected a significantly higher number of patients with recurrent HCC compared to multiphase enhanced CT (20 [100.0%] vs. 14 [70.0%]; P = .027). Furthermore, the additional use of Gd-EOB-DTPA-enhanced MRI led to the detection of 10 extra recurrent HCC lesions, with a median size of 1.2 cm (IQR 0.85, 1.43) (**Figure 1**). Based on the Gd-EOB-DTPA-enhanced MRI findings, the initial BCLC stage was upstaged in 2 (10%) patients (**Table 2**).

**Table 2:** Changes in BCLC staging with Gd-EOB-DTPA-enhanced MRI in the CT+MRI group.

			BCLC stage by Gd-EOB-DTPA-enhanced MRI			
	BCLC stage	Number	0	A	B	Changes in stage
BCLC stage by Multiphasic Enhanced CT	0	10(71)	9(64)	1(7)	0	1(7)
	A	4(29)	0	3(21)	1	1(7)

BCLC, Barcelona Clinic Liver Cancer.

All additionally detected lesions had a diameter of ≤1.5 cm and showed no arterial phase enhancement on multiphase enhanced CT. Hypointensity on the hepatobiliary phase of Gd-EOB-DTPA-enhanced MRI was the most sensitive feature for detecting these additional lesions (**Table 3**).

**Table 3:** Characteristics of the 10 additional recurrent HCC lesions detected by Gd-EOB-DTPA-enhanced MRI.

Characteristics	Nodules (n, %)
Arterial enhancement	7(70%)
Portal/Delayed phase hypointensity	9(90%)
Hepatobiliary phase hypointensity	10(100%)
T2 hyperintensity	9(90%)
Diffusion restriction	10(100%)
Imaging findings	Nodules (n, %)
≥1 cm showing arterial phase hyperenhancement and hypointensity in the portal venous or delayed phase.	3(30%)
≥1 cm showing arterial phase hyperenhancement, along with T2 hyperintensity, DWI hyperintensity, and hepatobiliary phase hypointensity	1(10%)
≥1 cm, showing T2 hyperintensity, hypointensity in the portal venous or delayed phase and hepatobiliary phase, as well as hyperintensity on diffusion-weighted imaging	3(30%)
<1 cm showing arterial enhancement and hypointensity in both portal/delayed and hepatobiliary phases	3(30%)
Total	10(100%)

### Diagnostic performance of blinded observers for HCC recurrence detection

**Table 4** and **5** show the diagnostic performance - including accuracy, sensitivity, specificity, positive predictive value (PPV), and negative predictive value (NPV) - of the two observers in the CT+MRI group for detecting intrahepatic HCC recurrence.

**Table 4:** Patient-based analysis of diagnostic performance in the CT+MRI group.

Observer	CT			MRI			*P value*
	Value	Tumors,n	95% CI	Value	Tumors,n	95% CI	
Observer 1							
AUROC	0.61		0.48-0.73	0.92		0.83-1.00	<0.001
Sensitivity	0.45	9/20	0.23-0.67	0.90	18/20	0.77-0.99	0.008
Specificity	0.76	13/17	0.56-0.97	0.94	16/17	0.83-1.00	0.248
Accuracy	0.59	22/37	0.44-0.75	0.92	34/37	0.83-1.00	0.001
PPV	0.69	9/13	0.44-0.94	0.95	18/19	0.84-1.00	0.132
NPV	0.54	13/24	0.34-0.74	0.89	16/18	0.75-1.00	0.021
Observer 2							
AUROC	0.69		0.65-0.93	0.90		0.80-0.99	0.002
Sensitivity	0.50	10/20	0.44-0.76	0.85	17/20	0.69-1.00	0.023
Specificity	0.88	15/17	0.74-0.97	0.94	16/17	0.83-1.00	1.000
Accuracy	0.68	25/37	0.63-0.84	0.89	33/37	0.80-0.99	0.024
PPV	0.83	10/12	0.66-0.96	0.94	17/18	0.84-1.00	0.709
NPV	0.60	15/25	0.53-0.81	0.84	16/19	0.68-1.00	0.081

PPV, positive predictive value; NPV, negative predictive value.

**Table 5:** Lesion-based analysis of diagnostic performance in the CT+MRI group.

Observer	CT			CT+MRI			*P value*
	Value	Tumors,n	95% CI	Value	Tumors,n	95% CI	
Observer 1							
AUROC	0.67			0.93			0.038
Sensitivity	0.50	9/20	0.27-0.73	0.92	18/20	0.81-1.00	0.007
Specificity	0.84	15/17	0.68-1.00	0.94	16/17	0.83-1.00	0.680
Observer 2							
AUROC	0.70		0.65-0.93	0.91			0.034
Sensitivity	0.50	10/20	0.27-0.73	0.88	17/20	0.74-1.00	0.021
Specificity	0.90	15/17	0.76-1.00	0.94	16/17	0.83-1.00	1.000

PPV, positive predictive value; NPV, negative predictive value.

In the patient-based analysis within the CT+MRI group (**Table 4**), Gd-EOB-DTPA-enhanced MRI demonstrated significantly higher AUROCs (Observer 1: 0.92; Observer 2: 0.90) compared to multiphase enhanced CT (Observer 1: 0.61; Observer 2: 0.69), with all comparisons being statistically significant (P <.05). The diagnostic sensitivity, accuracy, and inter-observer agreement (κ = 0.84) for MRI were also significantly superior to those for CT (P <.05). However, no significant differences were observed in specificity, PPV, or NPV between the two imaging modalities.

The lesion-based analysis demonstrated that in the CT+MRI group, Gd-EOB-DTPA-enhanced MRI showed significantly higher AUROC values and sensitivity compared to multiphasic enhanced CT, while no significant difference was observed in specificity (**Table 5**).

Comparison between the CT-alone and CT+MRI groups (**Table 6**) showed that the AUROC values for Gd-EOB-DTPA-enhanced MRI in the CT+MRI group (Observer 1: 0.92; Observer 2: 0.90) were likewise significantly higher than those for multiphasic enhanced CT in the CT-alone group (Observer 1: 0.71; Observer 2: 0.74). For both observers, the sensitivity of Gd-EOB-DTPA-enhanced MRI was also significantly superior to that of multiphasic enhanced CT in the CT-alone group. However, no significant differences were found in specificity, accuracy, negative predictive value, or positive predictive value.

**Table 6:** Patient-based intergroup comparison between CT and CT+MRI groups.

Observer	CT			CT+MRI			*P value*
	Value	Tumors,n	95% CI	Value	Tumors,n	95% CI	
Observer 1							
AUROC	0.71		0.57-0.84	0.92		0.83-1.00	<0.001
Sensitivity	0.56	10/18	0.32-0.79	0.90	18/20	0.77-0.99	0.027
Specificity	0.84	16/19	0.68-1.00	0.94	16/17	0.83-1.00	0.605
Accuracy	0.70	27/37	0.55-0.86	0.92	34/37	0.83-1.00	0.032
PPV	0.77	10/13	0.54-1.00	0.95	18/19	0.84-1.00	0.279
NPV	0.67	16/24	0.48-0.85	0.89	16/18	0.75-1.00	0.147
Observer 2							
AUROC	0.74		0.61-0.87	0.90		0.80-0.99	0.003
Sensitivity	0.56	10/18	0.32-0.79	0.85	17/20	0.69-1.00	0.046
Specificity	0.89	17/19	0.76-1.00	0.94	16/17	0.83-1.00	1.000
Accuracy	0.73	27/37	0.58-0.88	0.89	33/37	0.80-0.99	0.075
PPV	0.83	10/12	0.62-1.00	0.94	17/18	0.84-1.00	0.548
NPV	0.68	17/25	0.50-0.86	0.84	16/19	0.68-1.00	0.301

PPV, positive predictive value; NPV, negative predictive value.

In the patient-based analysis, excellent inter-observer agreement (κ = 0.84) was achieved for Gd-EOB-DTPA-enhanced MRI within the CT+MRI group, while good agreement (κ = 0.70) was observed for multiphasic enhanced CT in the same group. For the CT-alone group, multiphasic enhanced CT also demonstrated good inter-observer agreement (κ = 0.58).

## Discussion

In this study, we evaluated the incremental diagnostic value of Gd-EOB-DTPA-enhanced MRI in patients who had already undergone multiphase contrast-enhanced CT. Our findings clearly demonstrate that in HCC patients receiving curative MWA treatment, Gd-EOB-DTPA-enhanced MRI significantly improves diagnostic sensitivity for detecting occult intrahepatic recurrence compared to follow-up multiphase enhanced CT alone. Notably, this technique proves particularly accurate in identifying smaller (≤1.5 cm) recurrent lesions with atypical imaging features, which correlates with the detection of additional HCC nodules and subsequent modifications in HCC staging. Diagnostic performance analysis through intra-group paired comparisons revealed that Gd-EOB-DTPA-enhanced MRI exhibited significantly higher area under the receiver operating characteristic curve (AUROC) values and diagnostic sensitivity than multiphase enhanced CT in both patient-based and lesion-based analyses. These results substantiate the superior performance of Gd-EOB-DTPA-enhanced MRI over multiphase enhanced CT for post-MWA follow-up surveillance.

This finding carries important clinical implications, particularly given the high recurrence rate following curative ablation and the current lack of effective adjuvant therapies with proven long-term survival benefits (3, 4). Therefore, timely and accurate postoperative imaging surveillance constitutes an important approach influencing patients’ long-term survival outcomes. Our findings also possess practical significance, as smaller (≤1.5 cm) occult recurrent lesions with atypical imaging features represent an early disease stage (18–20) and remain amenable to curative-intent salvage therapies (such as repeat ablation or surgery), thereby enabling control of disease progression and improved outcomes (21–23).

Multiple meta-analyses have demonstrated that Gd-EOB-DTPA-enhanced MRI exhibits superior sensitivity and overall diagnostic accuracy compared to contrast-enhanced CT in detecting HCC nodules, primarily attributed to its enhanced capability in identifying HCC lesions smaller than 2 cm (24–26). Existing studies further indicate that utilizing Gd-EOB-DTPA-enhanced MRI as an additional imaging modality in patients initially diagnosed with solitary HCC by contrast-enhanced CT enables detection of additional nodules, leading to modifications in HCC staging and treatment strategies that ultimately improve patient outcomes (14, 27). Corroborating these findings, our study demonstrated that all HCC recurrent nodules missed by multiphasic enhanced CT were smaller than 1.5 cm, which were subsequently detected through the additional use of Gd-EOB-DTPA-enhanced MRI, resulting in modified HCC staging. However, while previous research has primarily focused on diagnosing primary HCC, studies investigating the evaluation of treatment response for post-operative HCC recurrence remain relatively limited.

Consistent with our results, a prospective study by Yasuharu et al (28) comparing the diagnostic performance of Gd-EOB-DTPA-enhanced MRI versus contrast-enhanced CT in evaluating HCC recurrence after RFA demonstrated that Gd-EOB-DTPA-enhanced MRI enabled earlier detection of smaller recurrent lesions (<2 cm) following curative RFA treatment. However, that study focused on HCC with typical recurrence patterns. Notably, compared to primary HCC, recurrent lesions after MWA often present with more complex imaging features. On one hand, the enhancement characteristics of HCC change with decreasing lesion size, and in the early tumor stage, the intratumoral neovascular architecture is immature and fragile (29). More importantly, the destruction and remodeling of the original vascular network by ablation therapy, along with the effects of ablative thermal stress on tumor cell biology and the altered microenvironment, collectively lead to aberrant angiogenesis patterns. This makes it difficult for these lesions to exhibit typical enhancement features on conventional contrast-enhanced imaging, thereby hindering reliable detection. As demonstrated by our results, all HCC recurrent nodules missed by multiphasic CT exhibited atypical enhancement features, such as absence or inconspicuousness of arterial phase enhancement. Excluding such lesions with atypical features could lead to an underestimation of the sensitivity of Gd-EOB-DTPA-enhanced MRI. Consequently, some studies have begun to explore the advantages of multi-sequence MRI (30–32).

Particularly in the hepatobiliary phase (HBP), normal hepatocytes demonstrate high signal intensity due to uptake of Gd-EOB-DTPA, while HCC cells exhibit low signal intensity owing to their impaired ability to uptake the contrast agent (33). This contrast significantly improves the detection rate and diagnostic sensitivity for HCC lesions. In an analysis of 111 atypical nodules by Golfieri et al (34), HBP hypointensity alone identified the majority of HCC lesions with atypical enhancement patterns (sensitivity: 88%; specificity: 97%), outperforming other imaging characteristics. Similarly, Choi et al (35) demonstrated in a retrospective study that HBP hypointensity showed the highest diagnostic sensitivity (99.5%) among all imaging features evaluated. On the other hand, multiple studies have utilized diffusion-weighted imaging (DWI), which is independent of lesion vascularity and directly reflects changes in cellular density, demonstrating high sensitivity (95.2%) in differentiating HCC from benign lesions (36–38). Building on these findings, Wei et al. (15) established novel diagnostic criteria using hepatocyte-specific contrast agents in a prospective study, proposing that the combination of HBP hypointensity and DWI hyperintensity can serve as diagnostic evidence for HCC, achieving high diagnostic sensitivity (93.71%). Our study similarly confirms that, in blinded evaluations, low HBP signal on Gd-EOB-DTPA-enhanced MRI was the most sensitive sign for detecting recurrent lesions missed by CT. However, although low HBP signal has high diagnostic sensitivity for HCC, it can also be seen in some benign lesions (such as dysplastic nodules, hemangiomas, or inflammatory pseudotumors). Therefore, low HBP signal is more useful for detecting HCC rather than for differential diagnosis. In contrast, DWI offers greater specificity for malignant nodules. Its “target sign” feature is particularly helpful in excluding intrahepatic cholangiocarcinoma, thereby enhancing the diagnostic specificity for HCC. It is worth emphasizing that T2-weighted imaging provides crucial auxiliary and complementary information. A definite high T2 signal helps confirm a lesion as a solid mass (thus excluding transient perfusion abnormalities) and provides reliable anatomical context. In the diagnostic performance analysis phase of this study, using high T2 signal, low HBP signal, and high DWI signal as diagnostic criteria for intrahepatic HCC recurrence with atypical enhancement features, two blinded observers both demonstrated high sensitivity and overall accuracy, further validating the significant value of Gd-EOB-DTPA-enhanced MRI in detecting occult HCC recurrence.

This study has several limitations. First, it was conducted at a single center with a relatively small sample size, which may limit the generalizability of the findings and increase the risk of selection bias. Although the cohort provided adequate statistical power for the primary paired intra-group comparisons, the overall sample size may still be insufficient to support more robust subgroup analyses. Importantly, our results should be interpreted as preliminary evidence suggesting the potential value of Gd-EOB-DTPA–enhanced MRI in detecting occult recurrence. Larger, well-designed multicenter prospective studies are warranted to further validate our conclusions.

Second, this was a retrospective observational study. Although randomized controlled trials would provide the highest level of evidence, randomizing patients to multiphasic CT rather than gadoxetate-enhanced MRI may raise ethical concerns, given the established superiority of MRI for HCC evaluation. Thus, well-designed observational studies may represent a pragmatic and feasible approach for comparing these modalities.

Third, patient allocation was inherently non-random. Gd-EOB-DTPA–enhanced MRI was selectively performed in patients with high-risk features (e.g., multiple or large tumors), equivocal CT findings, or elevated AFP despite negative CT, and was never used as routine surveillance. While this reflects real-world practice in which MRI functions primarily as a problem-solving tool, it may intrinsically favor MRI in comparative analyses. We attempted to mitigate this bias using two strategies (1): primary paired comparisons within the CT+MRI group, enabling direct head-to-head assessment of contemporaneous CT and MRI; and (2) supplementary inter-group comparisons supported by comparable baseline characteristics and recurrence rates between groups (all P > 0.05). Importantly, our findings highlight the incremental diagnostic value of MRI primarily in high-risk or equivocal scenarios, rather than supporting routine MRI surveillance for all patients. Given the greater resource requirements of gadoxetate-enhanced MRI, including cost, examination time, and limited availability, a risk-stratified strategy may be more practical. Specifically, MRI could be prioritized for patients with (1) elevated AFP despite negative CT findings (2), high-risk baseline tumor characteristics, or (3) indeterminate CT results. Such an approach may maximize the clinical benefit of MRI for detecting small (≤1.5 cm) occult lesions while maintaining real-world feasibility.

Fourth, our analysis was limited to CT and Gd-EOB-DTPA–enhanced MRI, without inclusion of other potentially valuable modalities such as contrast-enhanced ultrasound (CEUS). CEUS provides real-time assessment of lesion vascularity without radiation exposure, is widely available, and may be useful for characterizing small lesions or guiding biopsy and repeat ablation. Prior studies have reported that CEUS may achieve comparable or superior sensitivity to CT for detecting post-ablation recurrence, particularly for lesions with atypical enhancement patterns. Future comparative effectiveness research should evaluate multiple surveillance strategies, including head-to-head comparisons of CT, MRI, and CEUS with blinded interpretation, sequential algorithms (e.g., CT first-line with MRI/CEUS as problem-solving tools), and integration of emerging biomarkers for risk stratification.

In conclusion, this study demonstrates the superior efficacy of Gd-EOB-DTPA-enhanced MRI over multiphasic contrast-enhanced CT in detecting occult HCC recurrence, establishing it as a potentially more valuable follow-up modality after MWA. However, this conclusion should be interpreted in the context of our study population, where MRI was selectively performed for patients with high-risk features or inconclusive CT findings. Our results support the use of Gd-EOB-DTPA-enhanced MRI as a valuable adjunctive tool in this targeted population, enabling identification of additional HCC nodules and modification of initial HCC staging in a clinically significant proportion of patients. For routine surveillance of average-risk patients, contrast-enhanced CT remains an appropriate first-line modality, with MRI reserved for equivocal or high-risk cases. Future multicenter prospective studies with larger cohorts and predefined allocation criteria are warranted to validate these findings and establish optimal surveillance algorithms.

## Data Availability

The raw data supporting the conclusions of this article will be made available by the authors, without undue reservation.
